# Sequential chemoradiation in locally advanced head and neck cancer after induction chemotherapy: an induction chemotherapy schedule more suited to a limited resource setting

**DOI:** 10.3332/ecancer.2015.543

**Published:** 2015-06-04

**Authors:** Aparna Gangopadhyay, Partha Nath, Jaydip Biswas

**Affiliations:** Chittaranjan National Cancer Institute 37, S.P Mukherjee Road, Kolkata 700026, West Bengal, India

**Keywords:** chemoradiation, chemotherapy, head and neck cancer, limited resource healthcare

## Abstract

**Background:**

In our experience, induction docetaxel, platinum, and fluorouracil (TPF) chemotherapy and sequential chemoradiation in locally advanced head and neck cancer lowers compliance owing to their considerable toxicity. Most of our head and neck cancer patients have locally advanced disease at presentation. Physicians frequently prefer paclitaxel–cisplatin induction chemotherapy instead, because of better patient tolerance.

**Materials and methods:**

A total of 207 locally advanced head and neck cancer patients receiving paclitaxel and cisplatin prior to chemoradiation from November 2010 to October 2013 were studied retrospectively.

Parameters like febrile neutropaenia, treatment compliance, and response rates were compared to our institutional retrospective data with TPF chemotherapy. Response was assessed by Response Evaluation Criteria in Solid Tumours (Recist) version 1.1. Toxicity was assessed by Common Terminology Criteria for Adverse Events (CTCAE) version 4.0 during chemotherapy. Radiation Therapy Oncology Group (RTOG) acute toxicity criteria were used for assessment during chemoradiation.

**Results:**

Febrile neutropaenia with paclitaxel– cisplatin was significantly lower 3.4% (7/207) versus 44.5% (73/164) with TPF chemotherapy (two-tailed P value < 0.0001).

A 95.7 % (198/207) paclitaxel–cisplatin patients completed chemoradiation versus 87% with TPF. The difference was significant (two-tailed P value = 0.0070).

Response rate at treatment completion with paclitaxel –cisplatin was 89.7% versus 88% with TPF chemotherapy. No significant differences were observed (two-tailed P value = 0.7007).

**Conclusion:**

Induction paclitaxel and cisplatin with sequential chemoradiation in locally advanced head and neck cancer is more suitable in a limited resource setting. Lower toxicity, better compliance, and comparable response are encouraging in our study cohort.

## Background

Induction chemotherapy in locally advanced head and neck cancers prior to local therapy has been demonstrated to be non-inferior to concurrent chemoradiation in terms of overall survival (OS). Despite possible lack of survival advantage, downsizing of tumours, allowing organ preservation along with the possible benefit of eradication of micro metastases earlier in the course of therapy makes this a desirable approach for many head and neck oncologists worldwide [1].

Induction chemotherapy with TPF has gained popularity because of the edge they have in terms of disease response and possible survival benefit over other combinations that were in use earlier [2]. However, the debate on survival benefit continues [3, 4]. Recent studies reveal no significant benefit in OS with sequential chemoradiation following induction chemotherapy as opposed to concurrent chemoradiation alone for locally advanced head and neck cancer [5, 6, 7].

Although TPF is widely in use as the combination of choice for neoadjuvant chemotherapy in head and neck cancers, the incidence of toxicities remains considerable, and the supportive treatment required is often resource intensive. In view of limited resources in our setting, adequate and appropriate supportive care is not always affordable and available to all patients. In addition, nearly all patients presenting with locally advanced head and neck cancer in our scenario are from underprivileged sections of society with limited access to healthcare and have poor nutritional status. In terms of radiotherapy planning, adequate target coverage in locally advanced disease necessitates compromise in normal tissue sparing capabilities which worsens toxicity.

Consequently, the impact of treatment toxicity is considerable; it imposes a financial burden on the patient’s family and the healthcare system in general. The treatment interruptions that occur because of the toxicity also have a bearing on disease outcomes; the radiobiology of most head and neck tumours makes the issue of treatment gaps especially important in relation to tumour outcomes.

Retrospective data from 164 patients at our institute show that 32/164 (19.5%) patients who received induction TPF chemotherapy had no delays between chemotherapy cycles and sequential chemoradiation. It was initiated without undue delays in 49/164 (29.8%). Febrile neutropaenia occurred in 73/164 (44.5%) patients, with 100/164 (60.9%) patients able to undergo radical chemoradiation. Among those who underwent chemoradiation 87/100 (87%) completed the course of radiotherapy. The dropout rate from concurrent chemotherapy was high, with 29/100 (29%) patients being able to receive four cycles of concomitant chemotherapy. Unacceptable delays in chemoradiation owing to toxicity were noted in 46/100 (46%). According to our experience, sequential chemoradiation following induction TPF chemotherapy is not feasible in our limited resource setting.

Considering patients’ financial difficulties, poor nutritional status, and limited availability of supportive care resources, physicians often consider induction paclitaxel and cisplatin or carboplatin in locally advanced tumours after discussion with patients. In our experience patients tolerated a combination of paclitaxel and cisplatin or carboplatin fairly well. Toxicities are manageable on an outpatient basis and are not resource intensive.

This study aimed to compare induction paclitaxel and cisplatin chemotherapy to induction TPF in a limited resource scenario. Subsequent tolerance of sequential chemoradiation was also assessed. Any statistically significant differences in incidence of febrile neutropaenia, patient compliance with sequential chemoradiation, and disease response at completion of treatment were also assessed.

## Materials and methods

A total of 207 patients with inoperable locally advanced head and neck cancer who attended our institute from November 2010 to October 2013 were studied. Patients were considered inoperable either given the extent of disease or because of medical comorbidities not allowing surgery. A majority had a histological diagnosis of squamous cell carcinoma. All patients were with Eastern Cooperative Oncology Group (ECOG) performance status of either 0 or 1 (Table 1).

None of these patients had undergone any previous surgery, chemotherapy, or radiotherapy for their disease. All patients were assessed at the multidisciplinary board and considered for induction chemotherapy in view of huge tumour burden. Subsequent chemoradiation was considered by the board members given the medical comorbidities not allowing surgery.

After obtaining a histological diagnosis, pre treatment investigations included complete blood count, renal biochemistry, chest radiography, dental assessment, and local CT scan for tumour assessment.

### Induction chemotherapy

All patients had received at a dose of paclitaxel 175 mg/m^2^ and cisplatin 75 mg/m^2^ three weekly as per institutional protocol. Most patients (96.4%) received three cycles of induction chemotherapy. The remaining patients had four cycles of chemotherapy because of radiotherapy waiting times.

Toxicity related to chemotherapy was assessed at each visit prior to chemotherapy. Assessment of toxicity was performed according to CTCAE version 4. Delays in administration of chemotherapy because of toxicity were noted for all patients.

After completing three cycles of induction chemotherapy, tumour response was assessed at the department of head and neck oncology.

### Sequential chemoradiation

#### Conventional radiotherapy

A total of 90% patients received conventional radiotherapy. A dose of 66 Gy in 33 fractions over six-and-one-half weeks was prescribed. Conventional radiotherapy was delivered by 6 MV photons in 36% of these patients. The remaining patients were treated by telecobalt.

For all patients, the initial 44 Gy was delivered by lateral parallel opposed fields matched with a low neck anterior-posterior (AP) field; the off-cord fields were planned on completion of 44 Gy. Dose to the posterior neck was achieved by electron fields of appropriate size and energy.

#### Conformal radiotherapy and intensity-modulated radiotherapy (IMRT)

A total of 10% patients had undergone three dimensional conformal radiotherapy (3D CRT) or IMRT. These patients received up to 70 Gy in 35 fractions over seven weeks to the high risk planning target volume (PTV) and 54 Gy in 27 fractions over five-and-one-half weeks to uninvolved neck node levels.

All patients received concurrent weekly cisplatin at a dose of 30 mg/m^2^. Weekly complete blood counts and creatinine levels were monitored.

All patients were examined regularly for toxicity during the course of chemoradiation. Radiation toxicity was assessed according to RTOG acute toxicity criteria and recorded weekly.

The head and neck oncology department assessed every patient for disease response. Reassessment for tumour response at the department of head and neck oncology was done at three months of treatment completion.

#### Statistical analysis

Fisher’s two-tailed t-test was employed to test for significant differences in febrile neutropaenia, compliance to chemoradiation, and disease response at completion of treatment in patients who received paclitaxel–cisplatin induction chemotherapy to those who had received TPF induction chemotherapy. Statistical analysis was done using the GraphPad QuickCalcs Web site: http://www.graphpad.com/quickcalcs/contingency1 (accessed September 2014).

## Results

### Patient characteristics

Median age of patients was 54 years for males and 48 years for females, 72.6% of patients had ECOG performance status of 0. The remaining had a score of 1.

Among them 81% patients were males forming the larger part of the study group. Moderately differentiated squamous cell carcinoma was the commonest histological type of tumour, comprising 51.2% of all tumours. The commonest site of disease was hypopharynx constituting 44% of patients, followed by oropharyngeal tumours (35.7%). Laryngeal tumours formed 20% of the study cohort and consisted exclusively of males (Table 2).

All patients had large tumour burden with 50.2% patients in stage group IV and 49.8% in stage III (Table 3).

### Induction chemotherapy

All patients had three cycles induction chemotherapy; eight patients received four cycles owing to delays in starting radiotherapy because of long waiting times.

During induction chemotherapy 7/207 (3.4%) had febrile neutropaenia. In contrast, febrile neutropaenia incidence with TPF chemotherapy was 73/164 (44.5%). The difference was found to be statistically significant (two-tailed P value < 0.0001). The incidence of chemotherapy related toxicities were acceptable with 52 patients (25%) having mucositis of at least grade 1. No grade3 or 4 mucositis or neutropaenia was noted. No patients died while receiving chemotherapy. All patients started chemoradiation within an average of 4.5 weeks (range four to five weeks) of last chemotherapy.

Delays in administration of chemotherapy cycles were not noted beyond ten days in any patient.

A response rate of 89.2% was noted to induction chemotherapy, of which 83.8% was males and 16.4% were females. Complete remission (CR) was achieved in 7.2% patients, of which five patients progressed onto induction chemotherapy. Partial response (PR) occurred in 81.9%, of which 8.6% had stable disease.

**Figure 1.**
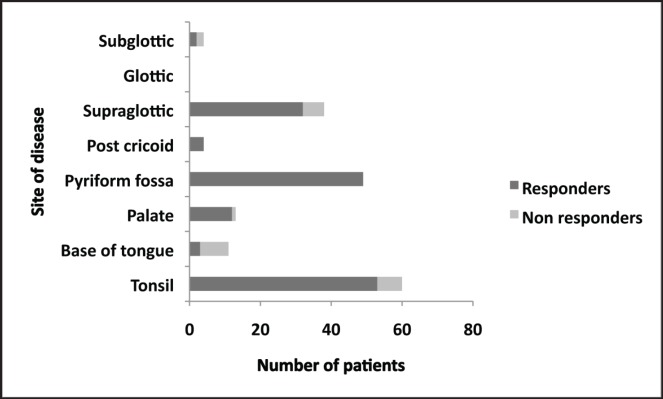
Proportion of responders to induction chemotherapy.

Patients with hypopharyngeal tumours were noted to have highest response rates of the primary site, whereas base of tongue tumours had lowest response (Figure 1).

### Sequential chemoradiation

All patients started chemoradiation within four to five weeks of completion of last cycle of induction chemotherapy. No delays in initiation of chemoradiation because of chemotherapy-induced toxicity were noted.

During chemoradiation, 23/207 (11.1%) patients needed hospitalisation for toxicity related causes. Among them 11 (5.3%) had febrile neutropaenia, 25/207 (12.1%) had grade 3 and 4 mucositis, with associated feeding difficulty, and two patients needed hospitalisation for an emergent tracheostomy. No treatment toxicity related deaths occurred. Toxicity related breaks in radiotherapy were noted in 17.6% patients.

Overall, 71 patients (34.3%) discontinued concurrent chemotherapy. All patients received at least three cycles of concomitant chemotherapy, with 87.9% completing four cycles. Among them 74.9% received up to the fifth cycle, and 66.7% patients had six cycles weekly of cisplatin during radiotherapy.

Among those patients who discontinued concurrent chemotherapy, 18 (25.4%) had rising creatinine, 29 (40.8%) had mucositis of grade 3 and 4, and the remainder refused to continue chemotherapy as the impaired quality of life was unacceptable for them. A total of 198/207 (95.7%) patients completed the course of radiotherapy. In comparison, 87/100 (87%) patients who had received TPF chemotherapy had completed their radiotherapy course. The difference was statistically significant with two-tailed P value = 0.0070.

At one month after completion of chemoradiation, the response rate was 89.7% with CR achieved in 85.8 %. In comparison, patients who received TPF had a response rate of 88%. No statistically significant difference in response rate was noted among patients’ at completion of chemoradiation irrespective of induction chemotherapy received. Two-tailed P value = 0.7007.

### Follow-up

At three months after treatment completion, CR was achieved in 90.9% patients, 17 patients (8%) had residual disease at three months of treatment completion, of which 60% had neck node disease only.

Median duration of followup was 26 months (range 3 to 40 months). Among them 22 patients were lost to follow-up. OS at 26 months was 75.1% (139/185). Progression-free survival at 26 months was 69.2% (128/185). Among those patients who experienced progression, one had distant metastases. Two patients were considered fit for salvage surgery and the remaining were referred for palliative care.

## Discussion

In a limited resource setting, most patients with head and neck cancer present at locally advanced stages. Induction chemotherapy is frequently administered in our scenario in view of perceived higher risk of distant metastases. Downsizing of tumours after induction chemotherapy often helps in improving normal tissue sparing during radiotherapy planning; conformal radiotherapy and IMRT facilities are not widely available making normal tissue sparing during chemoradiation difficult to achieve.

Patient cohorts in studies employing induction chemotherapy for locally advanced head and neck cancer comprised of a majority of oropharyngeal squamous cell carcinomas [6, 7]. In comparison, our study cohort had a large number of hypopharyngeal tumours. Locally advanced hypopharyngeal tumours are usually associated with greater tumour-related symptoms at presentation. Chemoradiation in hypopharyngeal tumours is associated with higher acute and late radiation toxicity. Interestingly, however, progression-free survival favours induction chemotherapy in non oropharyngeal tumours [7].

In our study, response rates to paclitaxel and cisplatin induction chemotherapy were encouraging. Considerable delays in the administration of induction chemotherapy and initiation of chemoradiation were not noted. Significantly lower incidence of febrile neutropaenia and better treatment compliance made induction of paclitaxel–cisplatin safer, more tolerable, and less resource intensive than TPF chemotherapy in our setting. The majority of patients continued with concurrent chemoradiation for the entire course in our study group and for those patients who discontinued concurrent chemotherapy, radiation toxicities were responsible in 40%.

No significant differences in response rates were observed among patients who received paclitaxel–cisplatin or docetaxel, cisplatin, and fluorouracil. Overall and progression-free survival among our cohort was found comparable with data available in literature. The results indicate that paclitaxel–cisplatin is more tolerable than TPF induction chemotherapy in a limited resource setting; sequential chemoradiation is more acceptable as well.

The benefits of induction chemotherapy in locally advanced head and neck cancer are questioned by results from recent studies indicating the lack of benefit in terms of OS with induction chemotherapy [7, 8]. Controversy exists, however, owing to limitations in these recent trials, which failed to clearly establish the lack of benefit from induction chemotherapy. An interesting result from a subset analysis of the DeCIDE trial was the lower number of distant metastatic events with induction chemotherapy, suggesting that it does eradicate micro-metastatic disease. Whether this may impact survival remains to be explored in future trials [9].

Functional organ preservation is one of the virtues of induction chemotherapy that has encouraged its use in locally advanced head and neck cancer prior to local therapy despite the risk of increased toxicity [10]. The strategy of chemo selection helps to reduce unwanted toxicity in these patients by identifying only those who derive benefit in terms of disease control and functional outcomes [11, 12]. Studies focusing on survival, disease control, and laryngeal–esophageal function after therapy are particularly relevant in regard to locally advanced head and neck cancer [13].

Since inception many induction chemotherapy regimens have been tried in head and neck cancer, but the regimens that incorporate a taxane have shown greatest clinical benefit [2]. However, the toxicity of the TPF regimen remains an area of concern, and hinders its clinical use in many patients. Herman *et al* in their study compared TPF to paclitaxel and carboplatin as induction therapy. They reported the latter combination was associated with superior locoregional control and progression-free survival [14].

Sanders *et al* found toxicity of induction TPF chemotherapy to interfere with subsequent sequential chemoradiation [15]. This is similar to our experience; sequential chemoradiation was poorly tolerated by patients who received TPF induction chemotherapy at our institute. TPF chemotherapy was associated with significantly higher febrile neutropaenia and reduced compliance to chemoradiation.

In a healthcare scenario like ours where patient affordability and limited resources are important factors considered at the time of treatment, a regimen associated with more toxicity causes hindrance to treatment schedules; less toxic regimens are useful in this regard. Delays and treatment gaps are of particular significance, especially in the context of head and neck cancer. A regimen that is better tolerated with the advantage of being more cost effective for patients and healthcare services, is of utmost importance in our scenario, and carries significance in the treatment of locally advanced head and neck cancer.

However, the results require further evaluation owing to a limited number of patients being studied at this time. Prospective studies including larger patient numbers are necessary in this regard. Also in our study, a majority of our patients received conventional radiotherapy. Whether these results would differ with the use of conformal techniques and IMRT remains to be explored.

## Conclusion

Paclitaxel and cisplatin induction chemotherapy is a more suitable alternative to the TPF regimen in locally advanced head and neck cancer in a limited resource healthcare scenario. Sequential chemoradiation after paclitaxel–cisplatin is well tolerated with acceptable disease control.

The findings of our study are particularly relevant to a limited resource setting as we are also aiming to reduce burden on healthcare services related to supportive care.

## Conflicts of interest

Authors declare no conflicts of interest.

## Figures and Tables

**Table 1.** Patient characteristics.

Patient characteristics (n = 207)		
**Gender**	Male	Female
**Total number**	168 (81.2%)	39 (18.8%)
**Median age (in yrs)**	54 years (range 24–67)	48 years (range 39–52)
**ECOG PS**		
**0**	91	31
**1**	77	8
**Histological diagnosis**		
**Poorly differentiated squamous cell carcinoma**	24	5
**Moderately differentiated squamous cell carcinoma**	79	27
**Well-differentiated squamous cell carcinoma**	65	7

**Table 2.** Sites of primary tumour.

Site	Male (n = 168)	Female (n = 39)	Total (n = 207)
**Oropharynx**	**38**	**36**	**74 (35.7%)**
**Tonsil**	31	23	60
**Base of tongue**	5	6	11
**Palate**	2	7	13
**Hypopharynx**	**88**	**3**	**91 (44%)**
**Pyriform fossa**	86	1	87
**Postcricoid**	2	2	4
**Larynx**	**42**	**0**	**42 (20.3%)**
**Supraglottic**	38	0	38
**Glottic**	0	0	0
**Subglottic**	4	0	4

**Table 3.** Stage and site of primary tumour.

Tumour stage and site	Hypopharynx (n = 91)	Oropharynx (n = 74)	Larynx (n = 42)	Overall
**IV**	49	24	31	(50.2%)
**III**	42	50	11	(49.8%)
